# Upregulation of miR-483-3p contributes to endothelial progenitor cells dysfunction in deep vein thrombosis patients via SRF

**DOI:** 10.1186/s12967-016-0775-2

**Published:** 2016-01-22

**Authors:** Lingshang Kong, Nan Hu, Xiaolong Du, Wenbin Wang, Hong Chen, Wendong Li, Sen Wei, Hao Zhuang, Xiaoqiang Li, Chenglong Li

**Affiliations:** Department of Vascular Surgery, The Second Affiliated Hospital of Soochow University, No.1055, Sanxiang Rd, Suzhou, 215000 Jiangsu China; Department of General Surgery, The Fourth Affiliated Hospital of Anhui Medical University, Hefei, 230022 China

**Keywords:** MiR-483-3p, Endothelial progenitor cells, Deep vein thrombosis, Serum response factor, Digital subtract angiography

## Abstract

**Background:**

Endothelial progenitor cells (EPCs) contribute to recanalization of deep vein thrombosis (DVT). This study aimed to detect miRNA expression profiles in EPCs from patients with DVT and characterize the role of miRNA in EPCs dysfunction.

**Methods:**

EPCs was isolated from DVT patients and control subjects, and miRNA expression profiles were compared to screen differential miRNAs. The candidate miRNAs were confirmed by RT-PCR analysis. The targets of miRNA were identified by bioinformatics analyses, luciferase reporter assay and gene expression analyses. The apoptosis, migration and tube formation of EPCs were examined by flow cytometry, transwell assay and matrigel tube formation assay. A rat model of venous thrombosis was established as in vivo model.

**Results:**

We identified miR-483-3p as a candidate miRNA upregulated in EPCs from DVT patients. By using miR-483-3p agomir and antagomir, we demonstrated that miR-483-3p decreased the migration and tube formation while increased the apoptosis of EPCs. Moreover, we identified serum response factor (SRF) as the target of miR-483-3p, and showed that SRF knockdown decreased the migration and tube formation while increased the apoptosis of EPCs. In addition, miR-483-3p inhibition led to enhanced ability of homing and thrombus resolution of EPCs in rat model of venous thrombosis.

**Conclusions:**

miR-483-3p is upregulated in EPCs from DVT patients, and it targets SRF to decrease EPCs migration and tube formation and increase apoptosis in vitro, while decrease EPCs homing and thrombus resolution in vivo. MiR-483-3p is a potential therapeutic target in DVT treatment.

**Electronic supplementary material:**

The online version of this article (doi:10.1186/s12967-016-0775-2) contains supplementary material, which is available to authorized users.

## Background

Deep vein thrombosis (DVT) occurs when a blood clot forms in the deep vein of human body, and is a common peripheral vascular disease that causes major morbidity and mortality in various medical conditions [1]. Moreover, lower and upper extremities DVT cause post-thrombotic syndrome (PTS) and pulmonary embolism (PE), which could result in over 15 % death rate in the first 3 month after diagnosis [2]. Currently, anticoagulation therapy is the standard method for DVT, but the failure to the removal of existing thrombus and the risk of PE hinder the use of the therapy [3]. Therefore, it is urgent to develop a safe and efficient therapy for DVT treatment.

Endothelial progenitor cells (EPCs), a bone marrow-derived circulating progenitor for the endothelial lineage, play an important role in pathological and physiological neovascularization in the adult [4, 5]. EPCs were recruited into the thrombus during a resolution and involved in thrombus recanalization [6–8]. Lower numbers of EPCs in the thrombus may result in diminished thrombus recanalization and resolution. Therefore, effective recruitment of EPCs into the thrombus may be a problem of clinical significance.

MiRNAs participate in various biological events [9, 10], and recent studies suggested that miRNAs are involved in EPCs function [11]. The upregulation of miR-107 in hypoxia condition led to EPCs differentiation inhibition by targeting HIF-1β [12]. miR-130a was involved in the regulation of autophagy function in EPCs via targeting Runx3 [13]. In addition, our previous studies showed that miR-150 and miR-126 contributed to EPCs function in vitro and improved thrombus recanalization and resolution in vivo [14, 15].

Here we reported the upregulation of miR-483-3p in DVT patients and demonstrated that ectopic expression of miR-483-3p attenuated the migration, tube formation and increased the apoptosis of EPCs via SRF in vitro. Moreover, we tested the efficacy of miR-483-3p modified EPCs in the treatment of vein thrombosis using a rat model.

## Methods

### Subjects

Eighty milliliter of peripheral blood were collected from DVT patients (n = 3) and control subjects (n = 3) at the Second Affiliated Hospital of Soochow University, Suzhou, China. The included DVT patients were confirmed by Color doppler ultrasound and lower extremity angiography without a history of hypertension, diabetes mellitus and other chronic diseases. Patients and healthy controls were matched by the age, gender and other risk factors (Table 1). The protocols were approved by the Institutional Review Board of the Second Affiliated Hospital of Soochow University and written informed consent was obtained from each participant.

**Table 1:** Baseline characteristics of DVT patients and healthy controls

	Control (n = 13)	DVT (n = 13)
Age, years (mean, SD)	55.4 ± 11.6	57.1 ± 13.2
Gender, females/males	7/6	7/6
Hypertension	0	0
Diabetes mellitus	0	0
Other chronic diseases	0	0

### Isolation of EPCs

EPCs were isolated and characterized according to previous methods [16, 17]. Peripheral blood mononuclear cells (PBMCs) were isolated using Ficoll-Isopaque Plus (Histopaque-1077; Sigma, MO, USA) gradient centrifugation method. The PBMCs were seeded onto fibronectin-coated cell culture flask, cultured in endothelial basal medium-2 (EBM-2; Lonza, MD, USA) supplemented with 20 % fetal bovine serum (FBS), vascular endothelial growth factor (VEGF; R&D Systems, MN, USA), human recombinant long insulin-like growth factor-1 (R3-IGF), ascorbic acid, and hydrocortisone and maintained at 37 °C, 5 % CO_2_ incubator. Medium change was performed after 4 days culture and early EPCs developed an elongated spindle-shaped morphology after 7 days culture. EPCs were characterized by confocal microscopy and flow cytometry. The cells were incubated with agglutinin 1 (FITC-UEA-1; Sigma Deisenhofen, Germany) and 1, 19-dioctadecyl-3, 3,3939-tetramethylindocar-bocyanine perchlorate (DiI)-labeled acetylated low density lipoprotein (Dil-Ac-LDL) as described previously [18]. Incorporation of DiI-Ac-LDL and binding of FITC-UEA-1 were detected under confocal microscope (Leica Microsystems GmbH, Germany). Cells with double positive staining of DiI-Ac-LDL and UEA-1 were identified as EPCs. The phenotypes of EPCs were analyzed for surface expression of CD34, CD133 and VEGFR-2 (all antibodies were purchased from MiltenyiBiotec, Bergisch, Germany). The third or fourth passages of EPCs were used.

### MiRNA expression profiles

Total RNA was extracted from EPCs using Trizol reagent (Invitrogen, CA, USA) according to the manufacturer’s instructions. MiRNA expression profiling was performed using a miRNA microarray analysis and miRNA array probes (LC Sciences) according to previous methods [19, 20]. For each sample, triplicates were analyzed and significant differences between DVT and healthy controls for a given detectable miRNA signal were calculated. The ratio of the two sets of detected signals (log2 transformed, balanced) and the P values for the t-test were calculated. The differentially detected signals with P < 0.01 were analyzed (Table 2).

**Table 2:** The primers of target sequences

Gene name	Primer usage	Sequence (5′–3′)
hsa-miR-199a-3p	RT primer	GTCGTATCCAGTGCGTGTCGTGGAGTCGGCAATTGCACTGGATACGACCTGCCC
	Forward primer	GGTGTCACTCCTCTCCTCC
	Reverse primer	CAGTGCGTGTCGTGGA
hsa-let-7e-5p	RT primer	GTCGTATCCAGTGCGTGTCGTGGAGTCGGCAATTGCACTGGATACGACAACTAT
	Forward primer	GGGGTGAGGTAGGAGGTTGT
	Reverse primer	GTGCGTGTCGTGGAGTCG
hsa-miR-483-3p	RT primer	GTCGTATCCAGTGCGTGTCGTGGAGTCGGCAATTGCACTGGATACGACAAGACG
	Forward primer	GGTGTCACTCCTCTCCTCC
	Reverse primer	CAGTGCGTGTCGTGGA
U6 (human)	RT primer	CGCTTCACGAATTTGCGTGTCAT
	Forward primer	GCTTCGGCAGCACATATACTAAAAT
	Reverse primer	CGCTTCACGAATTTGCGTGTCAT
hsa-miR-199a-3p	Agomir: sense	ACAGUAGUCUGCACAUUGGUUA
	Agomir: antisense	ACCAAUGUGCAGACUACUGUUU
	Antagomir: sense	UAACCAAUGUGCAGACUACUGU
hsa-let-7e-5p	Agomir: sense	UGAGGUAGGAGGUUGUAUAGUU
	Agomir: antisense	CUAUACAACCUCCUACCUCAUU
	Antagomir: sense	AACUAUACAACCUCCUACCUCA
hsa-miR-483-3p	Agomir: sense	UCACUCCUCUCCUCCCGUCUU
	Agomir: antisense	GACGGGAGGAGAGGAGUGAUU
	Antagomir: sense	AAGACGGGAGGAGAGGAGUGA
rno-miR-483-3p	Overexpression	GGAUCCGCACUCCUCCCCUCCCGUCUUGUCUGAGAAACAAGACGGAAGGAAACGUCACUUUUUUGAAUUC
rno-miR-483-3p sponge	Sponge	GGAUCCACAAGACGGGCCCGAGGAGUGCGAUACAAGACGGGCCCGAGGAGUGACCGGUACAAGACGGGCCCGAGGAGUGUCACACAAGACGGGCCCGAGGAGUGUUUUUUGAAUUC
rno-miR-483-3p	Forward primer	GCTGACTCACTCCTCCCCTC
	Reverse primer	TATGGTTGTTCACGACTCCTTCAC
U6 (rat)	Forward primer	ATTGGAACGATACAGAGAAGATT
	Reverse primer	GGAACGCTTCACGAATTTG
SRF (human)	Forward primer	GAGATCGGTATGGTGGTCGG
	Reverse primer	GTCAGCGTGGACAGCTCATA
SRF siRNA (human)	Sense	GCAUCAUGAAGAAGGCCUAUU
	Anti-sense	UAGGCCUUCUUCAUGAUGCUU
β-actin (human)	Forward primer	ACATCCGCAAAGACCTGTAC
	Reverse primer	GCCATGCCAATCTCATCTTG
SRF (rat)	Forward primer	ACCAGCTTCACTCTCATGCC
	Reverse primer	TGCATGGGGACTAGGGTACA
β-actin (rat)	Forward primer	ACCCGCGAGTACAACCTTC
	Reverse primer	ATGCCGTGTTCAATGGGGTA

### MiRNA quantitative real time RT-PCR analysis

Quantitative real time PCR was carried out using a Roche Light Cycler 480 (Roche, Switzerland), miRNA Qqcr Quantitation Kit (GenePharma, Shanghai, China), and 200 ng total RNA. RNA was transcribed into cDNA Synthesis Kit (Thermo Scientific, MA, USA). Expression of the U6 RNA was assessed as an internal control.

### Agomir and antagomir transfection

To overexpress and knockdown miR-483-3p, agomir and antagomir of miR-483-3p were transfected into EPCs using Lipofectamine 3000 (Invitrogen, CA, USA) according to the manufacturer’s instructions. Cells were collected at 48 h after transfection, and the expression of miRNA was confirmed by real time RT-PCR.

### Migration assay

Migration assay was performed in a transwell system as described previously. EPCs (2 × 10^5^ cells) were resuspended in serum-free EBM-2 medium and seeded on the upper chamber. The lower chamber was filled with EBM-2 medium supplemented with 20 % FBS. After incubation at 37 °C, 5 % CO_2_ for 24 h, the cells were stained with hematoxylin and the cell number in each well was counted in three randomly picked fields (200× magnification) under a light microscope. All the experiments were performed in triplicate.

### Tube formation assay

In vitro matrigel tube formation assay was performed to determine the angiogenic activity of EPCs as previously described [18]. Briefly, EPCs (5 × 10^4^ cells) were transfected with miR-483-3p agomir or antagomir and then seeded onto matrigel coated 48-well plate, and tubular structures of EPCs in the matrigel were evaluated under microscope after 24 h incubation. To quantify the length of newly formed tubes, six random phase contrast photos per well were taken, and the length of each tube was measured using Image J software. Tube length obtained from miR-483-3p antagomir transfected cells was set to 100.

### Apoptosis analysis by flow cytometry

EPCs were stained with annexin V and propidium iodide (PI) (eBioscience, CA, USA), and analyzed by flow cytometry as previously described [18].

### Luciferase assays

The pMIR-SRF-3′ UTR plasmid containing the putative binding site of the SRF 3′UTR downstream of the firefly luciferase gene was generated by cloning and inserting of a 395 bp sequence located at 3′UTR downstream into the SpeI and HindIII sites of the pMIR-REPORT Luciferase vector (Ambion, TX, USA). For luciferase activity measurement, HEK293 T cells were grown in 24-well plates until 60–70 % confluence and co-transfected with 100 ng Luciferase plasmid and 50 ng Renilla plasmid (Ambion) as a control for transfection efficiency, along with 650 ng miR-483-3p agomir or antagomir or negative control. The activity of Luciferase and Renilla was assessed after 48 h with the Dual Luciferase Reporter 1000 Assay System (Promega, WI, USA).

### Western blot analysis

EPCs were lysed in RIPA buffer followed by high-speed centrifugation. Cellular proteins were quantified using bicinchoninic acid, separated by sodium dodecyl sulfate–polyacrylamide gel electrophoresis and transferred onto polyvinylidenedifluoride membranes. After blocking, the membranes were incubated with the antibody for serum response factor (SRF) monoclonal (Cell Signaling Technology, MA, USA) or Actin (Sigma, MO, USA). Next the membranes were incubated with horseradish peroxidase-conjugated secondary antibodies and protein bands were detected with SuperSignal West Pico Chemiluminescent Substrate (Pierce, Rockford, IL) on x-ray films (Kodak, Tokyo, Japan).

### Lentivirus, vector production and cells transduction

The lentivirus, vector production and cell transduction were performed as previously described [21–23]. Partial pre-miR-483 sequences flanked by EcoRI and AgeI restriction site were constructed into the H1 promoter of lentivirus infectious virions pGLV3-H1-GFP-Puro (GenePharma, Shanghai, China). EPCs were infected with the viruses and selected in the presence of 5 μg/ml puromycin (Sigma), and the green fluorescent protein (GFP) signal of the infected cells was detected under microscope, and the expression of the miR-483 cluster in EPCs was measured by quantitative real-time PCR. To generate miR-483-3p sponge lentivirus virion, the sequences to miR-483-3p with mismatches at position 9–12 were constructed into the pGLV3-H1-GFP-Puro lentivirus infectious virions and used to infect EPCs.

### Rat EPCs isolation

Male Sprague–Dawley (SD) rats weighted 300 g were purchased from SLAC Experimental Animal Company (Shanghai, China). Isolation of rat EPCs was performed as previously described [7, 15, 24]. Bone marrow was harvested from both femurs and tibias of SD rats. Density gradient centrifugation with Ficoll-paque (GE Healthcare, Piscataway, NJ, USA) was used to isolate mononuclear cells. The cells were seeded into flask pre-coated with human fibronectin (Sigma) and cultured in EGM-2-MV (Lonza, MD, USA) medium at 37 °C in a 5 % CO_2_ incubator. After 4 days, non-adherent cells were removed by PBS washing, and the adherent cells were cultured with fresh medium. The third and fourth generations of EPCs were used.

### Rat model of venous thrombosis

The animal protocol was approved by the Institutional Animal Care and Use Committee of Soochow University. SD rats were purchased from the Experiment Animal Center of Soochow University. The rats were anesthetized by intraperitoneal injection of 10 % chloral hydrate. A midline laparotomy was performed. The infrarenal inferior vena cava (IVC) was exposed and all side branches were ligated with 7-0 Prolene suture. The posterior venous branches were blocked by electric coagulation. A 7-0 Prolene suture was tied down on the IVC just below the left renal vein. At the same time, a microvascular clamp was attached to the confluence of iliac veins for 15 min to block the blood flow and induced the thrombus in IVC. The skin was sutured and the rats were allowed to recover after the surgery. Then the rats were divided into four groups for cell transplantion via tail intravenous injection (n = 10): (A) blank control group (blank control) received same volume of cell culture medium; (B) EPCs/pGLV3-H1-GFP-Puro vector group (EPCs/vector) received 1.0 × 10^6^ EPCs transfected with pGLV3-H1-GFP-Puro control vector; (C) EPCs/pGLV3-H1- GFP-Puro-miR-483-3p group (EPCs/miR- 483-3p) received 1.0 × 10^6^ EPCs transfected with pGLV3-H1-GFP-Puro-miR-483-3p; (D) EPCs/pGLV3-H1-GFP-Puro-miR-483-3p sponge group (EPCs/miR-483-3p sponge) received 1.0 × 10^6^ EPCs transfected with pGLV3-H1-GFP-Puro-miR-483-3p sponge.

### Histology

Seven days after the injection of either EPCs or control medium, the animals were sacrificed and IVC segments containing the thrombus were carefully harvested, and excess blood was absorbed with filter paper. The thrombi were weighed and embedded in optimal cutting temperature (OCT) media and flash-frozen in a −80 °C. Five micrometer cryosections were cut for histological analyses and a confocal microscope (Leica Microsystems) was used to analyze the homing of EPCs. The remaining samples were used for the analysis of thrombus organization and recanalization with hematoxylin and eosin (HE) staining.

### DSA

IVC venography was performed with digital subtract angiography (DSA, GE Innova 3100, USA) by injecting contrast media into rat caudal vein or femoral vein to determine the recanalization and resolution of thrombus in vivo.

### Statistical analyses

All statistical analyses were carried out using SPSS v21 (SPSS, Chicago, IL). Data are presented as mean ± SEM. Student’s t-test or one way ANOVA followed by Least Significant Difference test was used to compare the differences between or among the groups. Univariate comparisons of two independent groups were done using the Mann–Whitney-U test. Comparisons of multiple groups were performed with the Kruskal–Wallis test. P < 0.05 was considered significant.

## Results

### The upregulation of miR-483-3p in EPCs from patients with DVT

**Fig. 1:**
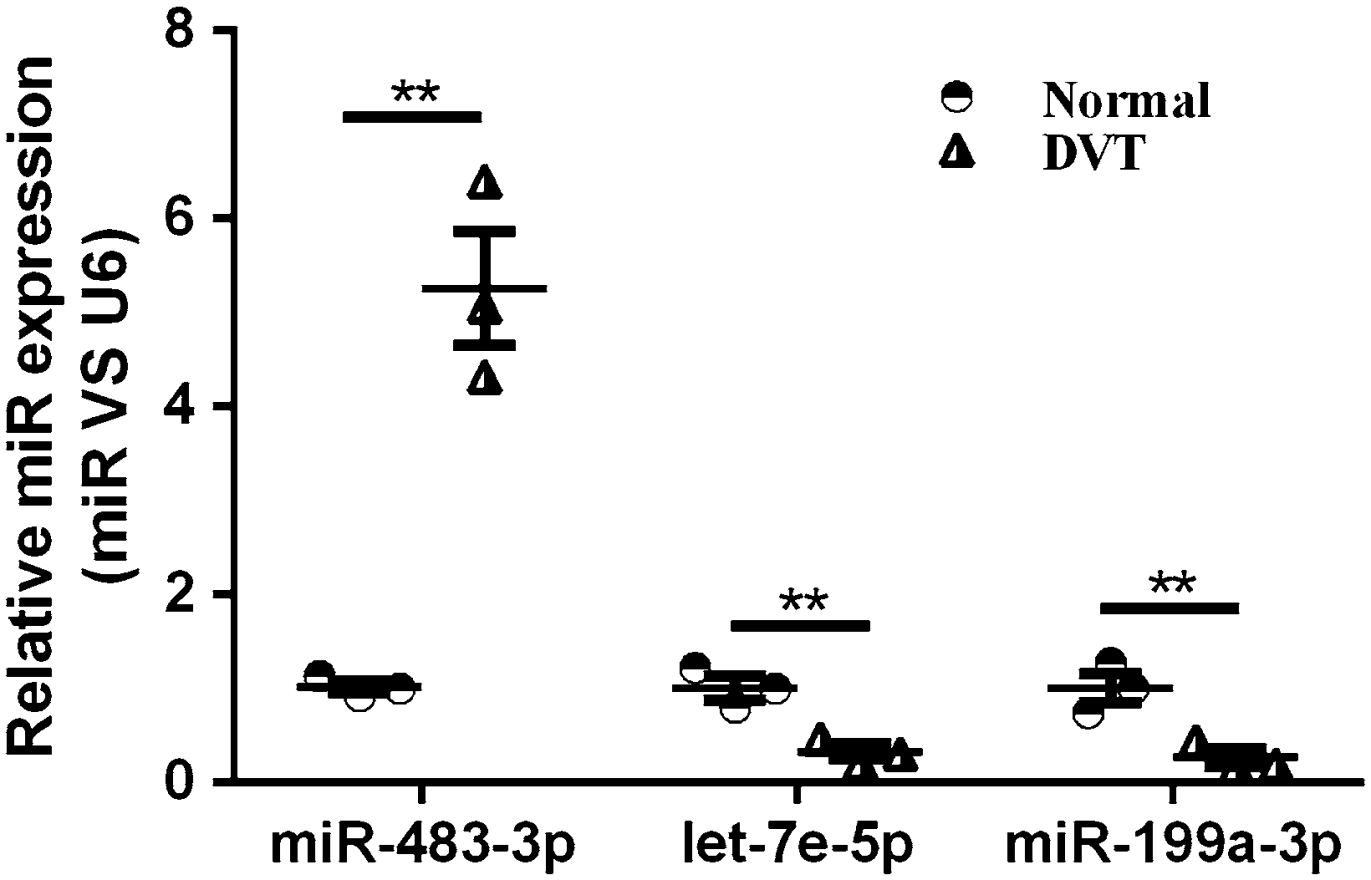
Differentially expressed miRNAs in EPCs from healthy control and patients with DVT by microarray (Arraystar, human miRNA 18.0 chip). Three aberrantly expressed miRNAs were identified by quantitative real-time PCR. Data are expressed as mean ± SEM (n = 3, ^**^P < 0.01 vs. health control)

EPCs were isolated from both DVT patients (n = 3) and healthy donors (n = 3), and the expression of miRNA was determined using a microarray assay (Additional file 1: Figure S1). Three miRNAs (upregulated miR-483 and downregulated let-7e and miR-199a) exhibited significantly fold changes (Fig. 1). To further confirm the accuracy of the microarray data, we performed quantitative RT-PCR to evaluate the expression of miRNA in DVT (n = 3) and healthy control (n = 3). Our results showed that the expression of miR-483-3p was significantly upregulated (Fig. 1, fivefold increase, P < 0.05), indicating the potential role of miR-483-3p in DVT.

**Fig. 2:**
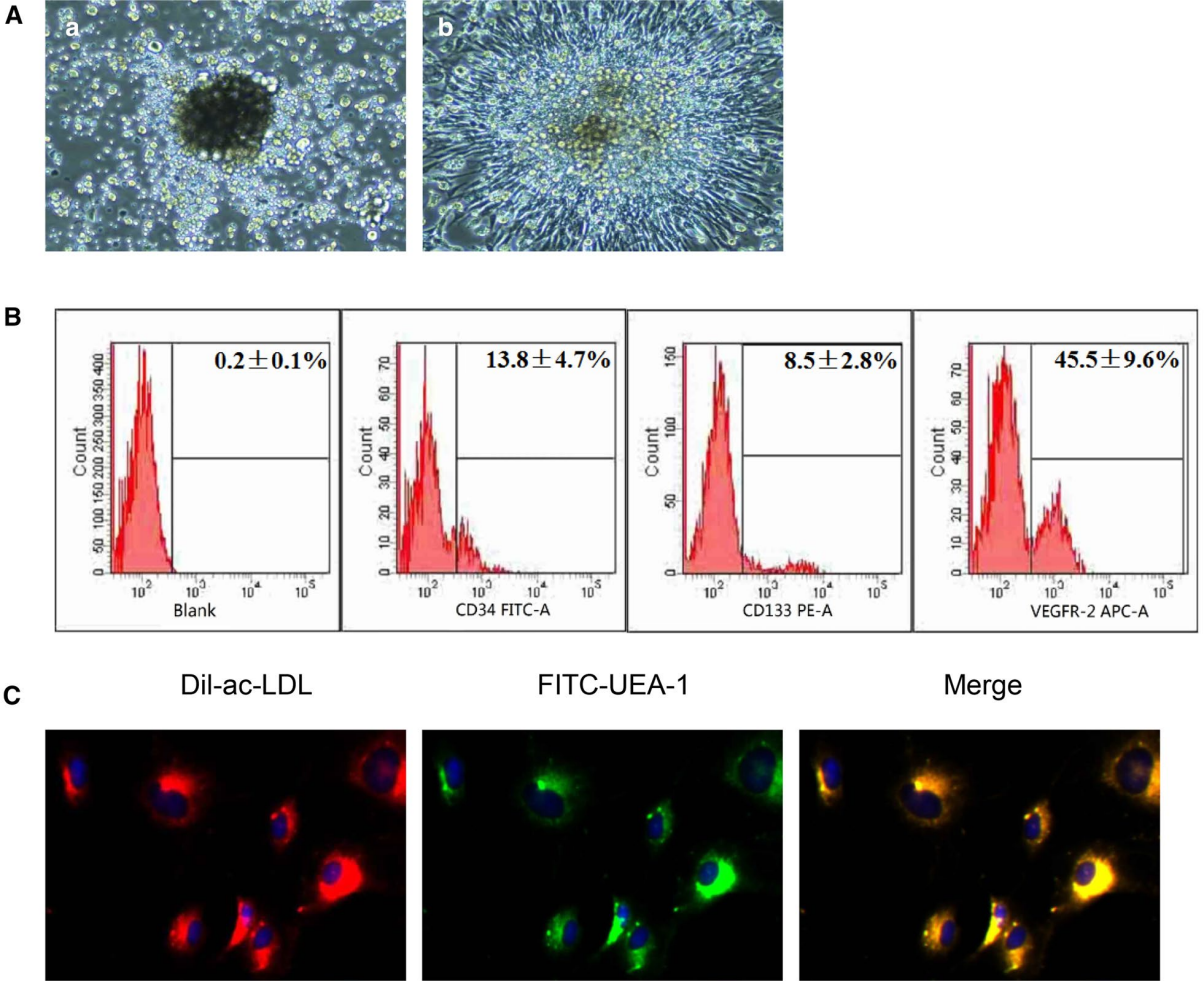
Isolation and characterization of EPCs. **A** On day 3 (**a**), EPCs colony exhibited a round morphology and formed a central cluster. On day 7 (**b**), EPC exhibited spindle-shaped cell radiating from the central cluster (×100). **B** FACS analysis of the cell surface markers of EPCs at day 14 (CD34, CD133, VEGFR-2). Data are expressed as mean ± SEM.** C** Staining of DiI-Ac-LDL, FITC-UEA-1, and merged image of double staining of FITC-UEA-1 and DiI-Ac-LDL (×400)

EPCs colony exhibited a round morphology and formed a central cluster after 3 days of culture (Fig. 2Aa). An elongated spindle-shaped morphology was found in early EPCs after seventh day of culture (Fig. 2Ab). FACS analysis showed the expression of CD34, CD133 and VEGFR-2 on EPCs at day 14 (Fig. 2B). EPCs were further characterized by double staining with functional marker FITC-UEA-I and Dil-ac-LDL (Fig. 2C).

MiR-483-3p decreased the migration, tube formation and increased the apoptosis of EPCs.

**Fig. 3:**
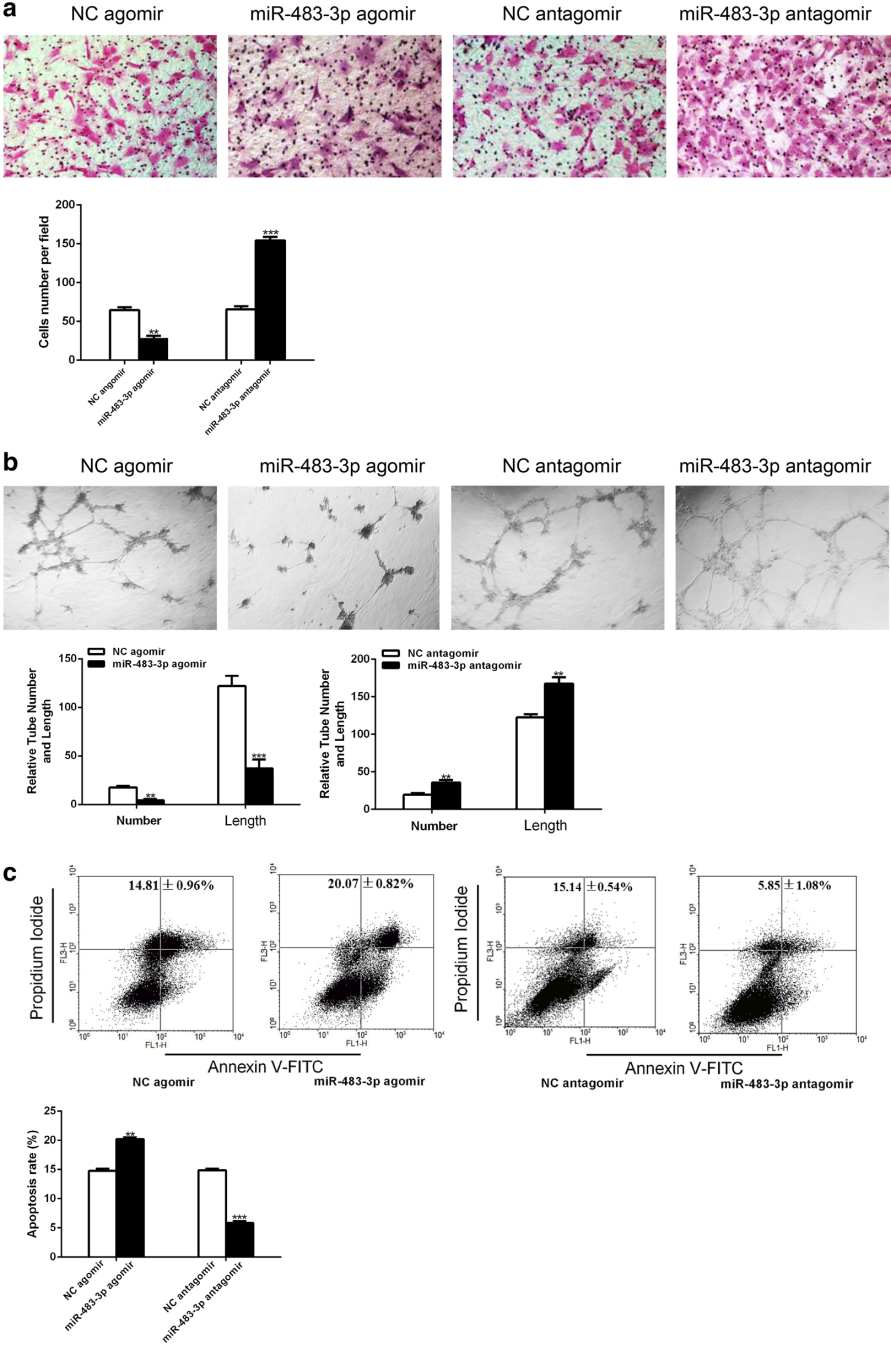
miR-483-3p regulates migration, tube formation, and apoptosis of EPCs. EPCs were transfected with miR-483-3p agomir or antagomir or negative control. **a** Migrated cell counting (×200). **P < 0.01 vs. negative control, ***P < 0.001 vs. negative control (n = 3). **b** Relative tube number and length (×100). **P < 0.01 vs. negative control, ***P < 0.001 vs. negative control (n = 3). **c** Cell apoptosis was determined by annexin V/PI staining. **P < 0.01 vs. negative control ***P < 0.001 vs. negative control (n = 3). All data are expressed as mean ± SEM

To clarify the role of miR-483-3p in EPCs, we employed miR-483-3p agomir and antagomir to overexpress and inhibit miR-483-3p in EPCs. EPCs transfected with miR-483-3p agomir displayed decreased ability of migration (Fig. 3a), tube formation (Fig. 3b) and increased apoptosis (Fig. 3c). EPCs transfected with miR-483-3p antagomir exhibited opposite behaviors on cell migration, tube formation and apoptosis (Fig. 3a–c).

### SRF is the target gene of miR-483-3p in EPCs

**Fig. 4:**
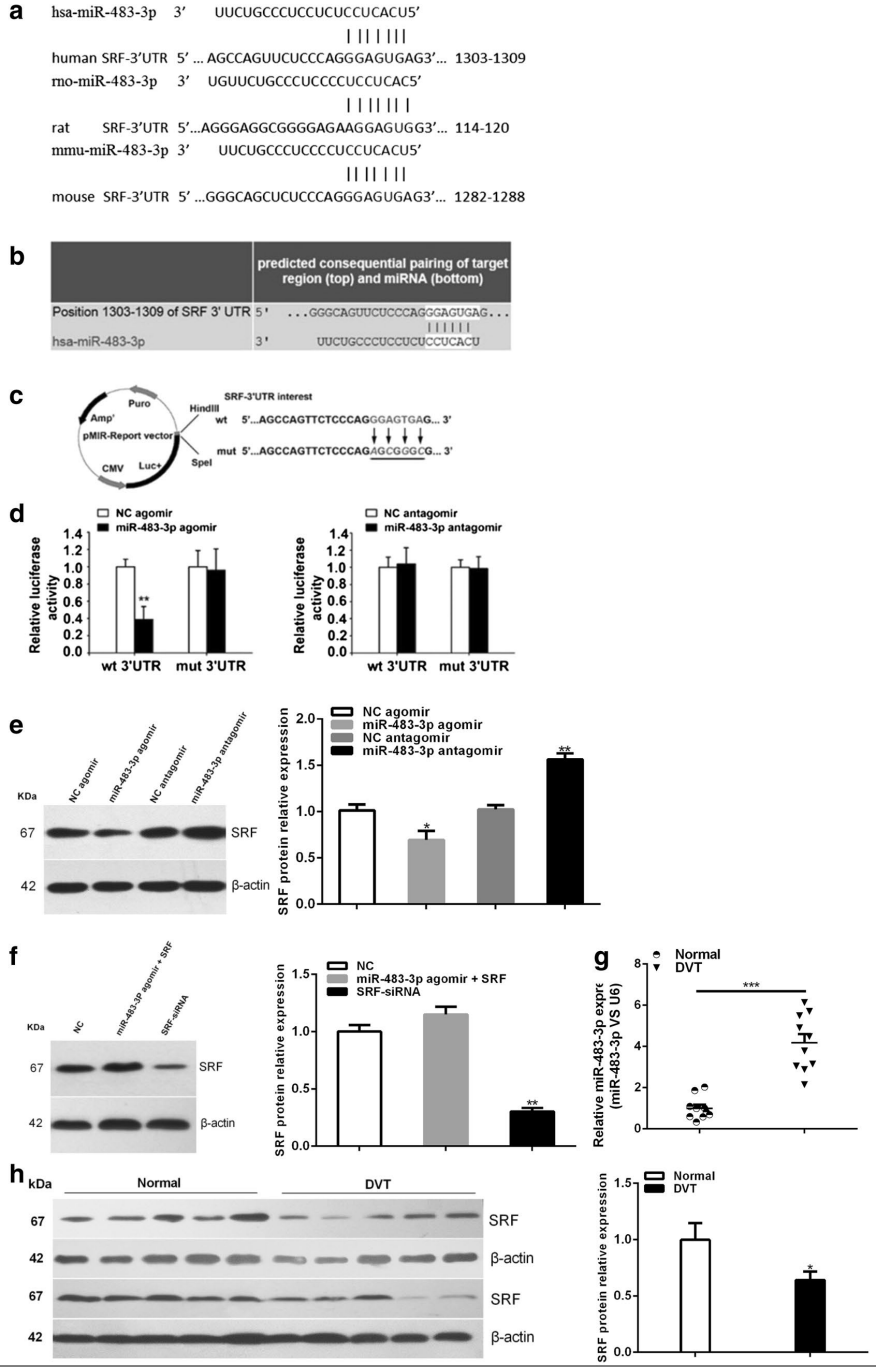
SRF is a validated target of miR-483-3p. **a** SRF is a putative target of miR-483-3p. Putative binding sites of miR-483-3p in the SRF 3′UTR predicted by TargetScan among different mammalian species. **b** Putative binding sites of hsa-miR-483-3p in the human SRF 3′UTR (*white sequences*) predicted by TargetScan. **c** Schematic graph of the constructed reporter plasmid containing putative binding sites of hsa-miR-483-3p in the SRF 3′UTR. SRF-3′UTR mut indicates the SRF-3′UTR with mutation in miR-483-3P-binding site. The mutated nucleotides in SRF-3′UTR fragments are *underlined*. **d** Dual luciferase report assays were performed on HEK 293 T cells. *Each bar* represents mean values ± SEM (n = 3, **P < 0.01). **e** The protein level of SRF in EPC transfected with miR-483-3p agomir and antagomir. *Each bar* represents mean values ± SEM (n = 3, *P < 0.05 vs. negative control; **P < 0.01 vs. negative control). **f** The protein level of SRF in EPC transfected with miR-483-3p agomir and SRF, SRF siRNA. *Each bar* represents mean values ± SEM (n = 3, **P < 0.01 vs. negative control). **g** miR-483-3p expression was measured in healthy control and patients with DVT by quantitative real-time PCR. Data are expressed as mean ± SEM (n = 10, ***P < 0.01 vs. health control). **h** The protein level of SRF in patients with DVT and health control. *Each bar* represents mean values ± SEM (n = 10, *P < 0.05 vs. health control)

Based on multiple databases (TargetScan, Microcosm Targets and RNA22-HAS), SRF was predicted to have a putative miR-483-3p binding sites within its 3′UTR (Fig. 4a, b).

To confirm that SRF is a direct target of miR-483-3p, we performed luciferase reporter assay and found decreased luciferase activity after a 395 bp region of 3′-UTR SRF was cloned into the luciferase report vector (Fig. 4c, d).

To confirm the ability of miR-483-3p to inhibit SRF expression, we transfected EPCs with miR-483-3p agomir and antagomir. EPCs transfected with miR-483-3p agomir exhibited significantly decreased SRF protein level while EPCs tranfected with miR-483-3p antagomir exhibited increased SRF protein level (Fig. 4e). As expected, we detected reduced protein level of SRF in EPCs after SRF siRNA transfection (Fig. 4f).

In addition, DVT patients (n = 10) and healthy control subjects (n = 10) were included to verify the upregulation of miR-483-3p in EPCs from DVT patients by quantitative real-time PCR (Fig. 4g). Western blot analysis showed decreased protein level of SRF in DVT patients (n = 10) compared to healthy controls (n = 10) (Fig. 4h).

### MiR-483-3p regulates EPCs function by targeting SRF

**Fig. 5:**
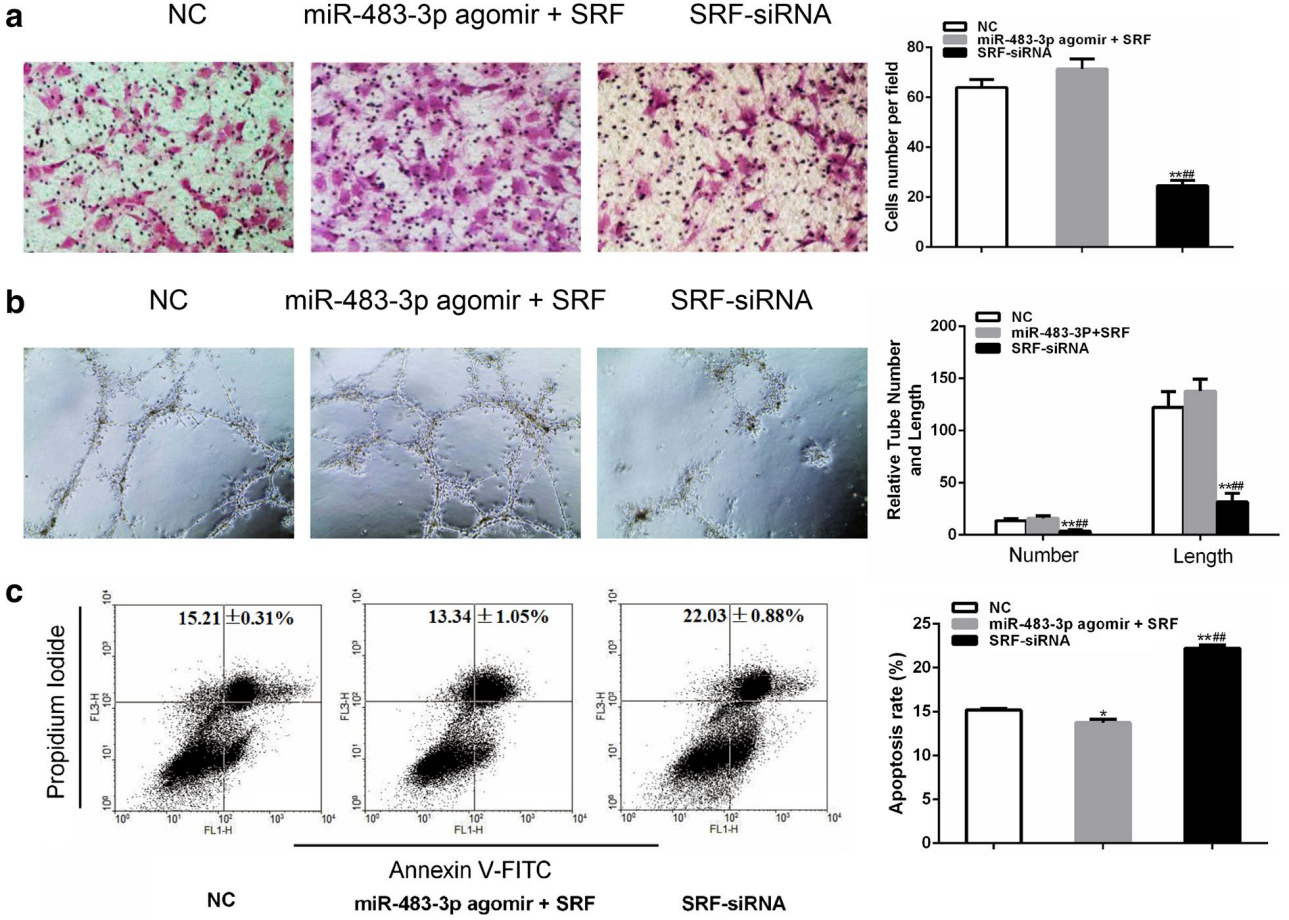
miR-483-3p and SRF functionally regulate EPCs function. **a** Decreased EPCs migration in EPCs transfected with SRF-siRNA. (n = 3, **P < 0.01 vs. negative control; ^##^P < 0.01 vs. miR-483-3p agomir + SRF). **b** Decreased tube number and length in EPCs transfected with SRF-siRNA (×100) (n = 3, **P < 0.01vs NC; ^##^P < 0.01 vs. miR-483-3p agomir + SRF). **c** Increased EPCs apoptosis after SRF-siRNA transfection. (n = 3, *P < 0.05 vs. negative control; **P < 0.01 vs. negative control; ^##^P < 0.01 vs. miR-483-3p agomir + SRF). *Each bar* represents mean values ± SEM

To explore the role of SRF in EPCs function, EPCs were transfected with SRF siRNA. EPCs transfected with SRF siRNA exhibited decreased cell migration (Fig. 5a), tube formation (Fig. 5b) and increased apoptosis (Fig. 5c), compared to EPCs transfected with vehicle controls. To further confirm that miR-483-3p modulated EPCs function by targeting SRF, we performed rescue experiments by co-transfection of miR-483-3p agomir and SRF expression vector in EPCs, and EPCs showed similar trend of cell migration, tube formation and apoptosis as controls (Fig. 5a–c).

### Knockdown of miR-483-3P in EPCs via lentivirus-mediated miR-483-3p sponge

**Fig. 6:**
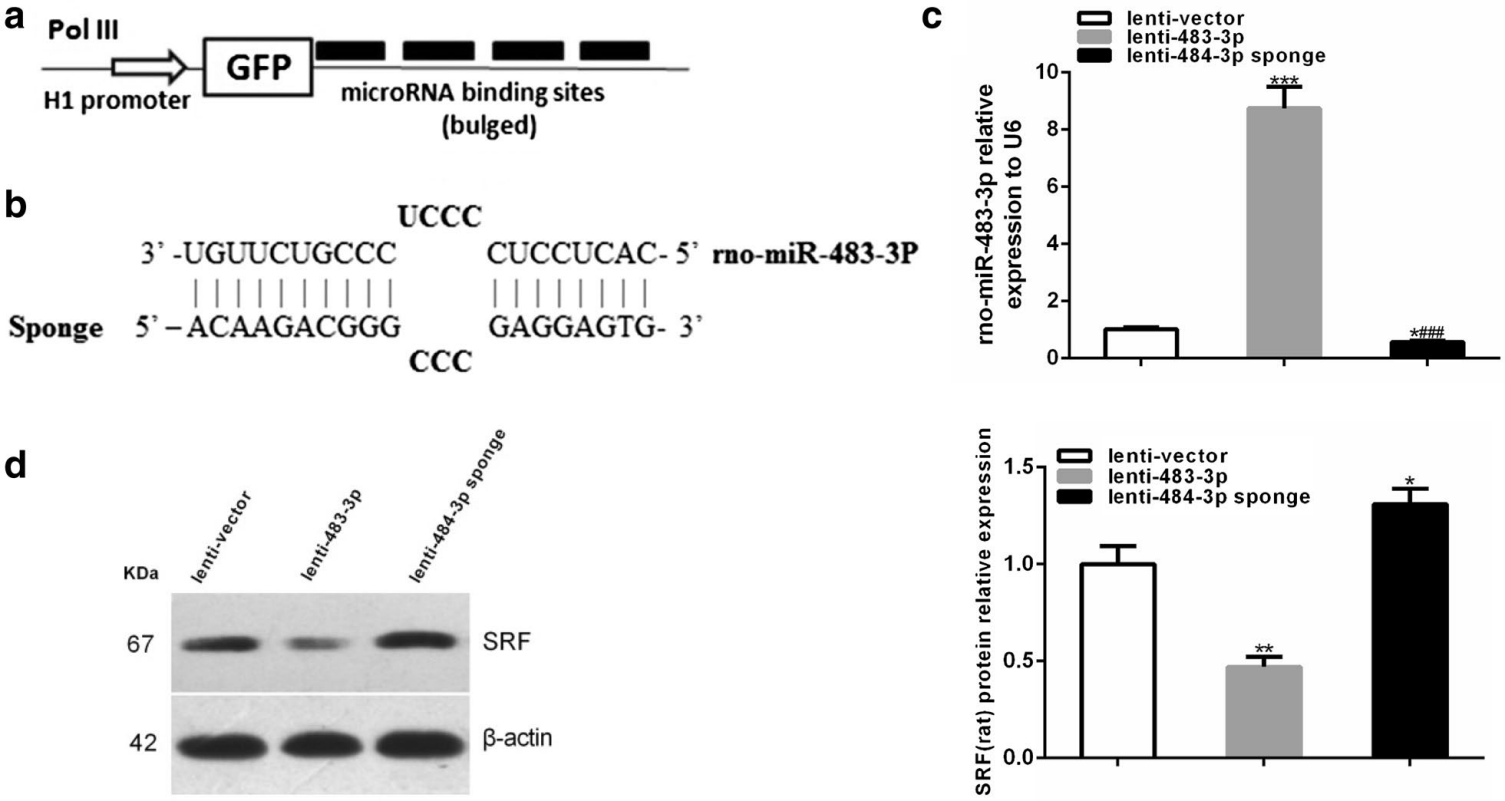
miR-483-3p sponge rescues SRF expression in EPCs. **a** Schematic graph of miRNA construction. **b** Binding sites of miR-483-3p sponge. **c** The relative expression of miR-483-3p after infection with lenti-vector control, lenti-483-3p and lenti-483-3p sponge. The relative expression data were normalized to the amount of U6, and expressed as mean ± SEM (n = 3, *P < 0.05 vs. lenti-vector; ***P < 0.001 vs. lenti-vector; ^###^P < 0.001 vs. lenti-483-3p). **d** SRF protein level was significantly increased after miR-483-3p sponge treatment. *Each bar* represents mean values ± SEM (n = 3, *P < 0.05 vs. lenti-vector; **P < 0.01 vs. lenti-vector)

We constructed a miR-483-3p sponge consisted of a decoy vector containing tandem repeated miRNA binding sites downstream of GFP (Fig. 6a). Binding sites for miR-483-3p were complementary in the seed region with a bulge at positions 9–12 to prevent RNA interference-type cleavage and degradation of the sponge RNA constructed into the pGLV3-H1-GFP-Puro lentivirus infectious virions (Fig. 6b). EPCs were infected with lentivirus-mediated vector control, miR-483-3p and miR-483-3p sponge, and the expression of the miR-483 cluster was measured by quantitative real-time PCR. MiR-483-3p sponge lentivirus virions totally silenced the expression of miR-483-3p (Fig. 6c). Western blot analysis showed that SRF protein level was significantly increased after miR-483-3p sponge treatment (Fig. 6d).

### MiR-483-3p inhibited EPCs homing

**Fig. 7:**
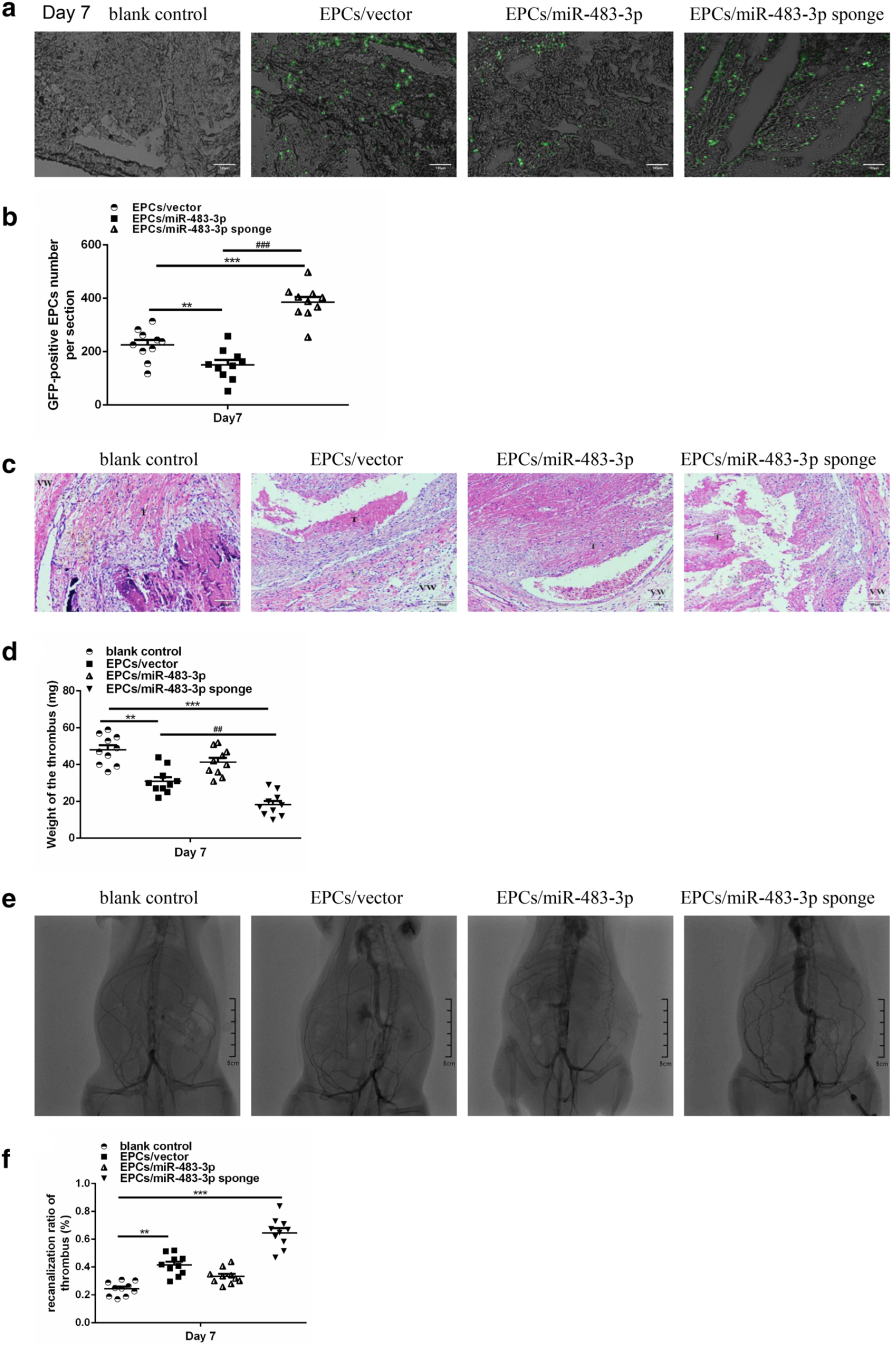
MiR-483-3p decreases EPCs homing, thrombus recanalization and resolution. **a** Representative images of recruitment of GFP-positive EPCs in the deep venous thrombosis (×200). **b** Quantification of GFP-positive EPCs in thrombus sections. Data are expressed as mean ± SEM (n = 10, **P < 0.01 vs. EPCs/vector; ***P < 0.001 vs. EPCs/vector; ^###^P < 0.001 vs. EPCs/miR-483-3p). **c** Hematoxylin and eosin (HE) staining of thrombus Sects. (200× magnification). *T* means thrombus and *VW* indicates venous wall. **d** Weight of the venous thrombi at day 7 post the transplantation. Data are expressed as mean ± SEM (n = 10, **P < 0.01 vs. blank control; ***P < 0.001 vs. blank control; ^##^P < 0.01 vs. EPCs/vector). **e** Significantly increase of thrombus recanalization and resolution was found in rats transplanted with EPCs transfected with miR- 483-3p sponge compared to EPCs transfected miR-483-3p on Day 7. **f** The recanalization of thrombus was quantified with Image J software by the area ratio of contrast agent in vascular with thrombosis. Data are expressed as mean ± SEM (n = 10, **P < 0.01 vs. blank control; ***P < 0.001 vs. blank control). Comparison among multiple groups was performed by one-way ANOVA followed by Least Significant Difference test

A rat model of DVT was established by inferior vena cava ligation. EPCs transfected with pGLV3-H1-GFP-Puro-miR-483-3p were intravenously administrated into the rats. Cyrosections of thrombus were prepared on day 7 after EPCs transplantation. EPCs were found to home and locate around the thrombus after transplantation. Significantly increased EPCs homing was found in the rats infused with EPCs/miR-483-3p sponge group compared to EPCs/miR-483-3p group on day 7 after EPCs transplantation (Fig. 7a, b).

### Inhibition of miR-483-3p promoted thrombus recanalization and resolution

HE staining showed that nucleated cells, including monocytes, endothelial cells and neutrophil granulocytes entered into the perimeter of the thrombus on day 7. The red blood cells, platelets and fibrin were dried red in the center of thrombus. There were more nucleated cells and channels in EPCs/vector and EPCs/miR-483-3p sponge group than in control group on day 7 (Fig. 7c). In addition, miR-483-3p sponge decreased the weight of thrombus (IVC + thrombus at harvest). Compared with control group, the weight of thrombus was similar to EPCs/miR-483-3p group, whereas significant decrease of thrombus weight was observed in EPCs/vector and EPCs/miR-483-3p sponge groups on day 7 after transplantation. Furthermore, the thrombus weight of EPCs/miR-483-3p sponge group was even lower than that of EPCs/vector group (18.7 ± 6.1 vs. 30.6 ± 8.3 mg on day 7, P < 0.05, Fig. 7d).

DSA has been widely accepted as the reference standard for DVT diagnosis and therefore we evaluated the thrombus recanalization and resolution in vivo by using DSA. A contrast agent was injected into the rats to illustrate the vessel. We observed significantly increased thrombus recanalization and resolution in rats transplanted with miR-483-3p sponge transfected EPCs compared to miR-483-3p transfected EPCs on day 7 (Fig. 7e, f).

## Discussion

In the present study, we reported several important findings on the role of miR-483-3p in EPCs. First, miR-483-3p expression was increased in EPCs derived from patients with DVT. Overexpression of miR-483-3p decreased migration and angiogenic potential of EPCs while increased apoptosis of EPCs. Moreover, we identified SRF as the target of miR-483-3p in EPCs. The migration, angiogenic potential and apoptosis of EPCs were positively regulated by SRF, and the suppression of SRF by miR-483-3p contributed to the effects of miR-483-3p on EPCs function. In addition, we established a rat model of vein thrombosis and observed that transplantation with EPCs transfected with miR-483-3p displayed increased homing ability and improved the thrombus recanalization and resolution in vivo. Taken together, our data suggest that miR-483-3p plays an important role in EPCs dysfunction, and upregulated miR-483-3p in EPCs from DVT contributes to impaired EPCs function, which is likely due to decreased SRF.

Vein thrombi resolution is a complex and spontaneous process that requires the orchestration of different cells [25]. Three main steps include the infiltration of inflammatory cell into the thrombus, tissue remodeling and angiogenesis. The vascularization of the thrombus is an early and important event on the efficacy of thrombus resolution [25]. Growing evidences have suggested that bone marrow-derived EPCs are recruited into the thrombus and involved in the thrombus organization and resolution [8, 26]. Several studies have reported the importance of EPCs in injured vessels repairmen and ischemic tissues revascularization [27]. We previously demonstrated that bone marrow-derived EPCs transplantation altered the vein microenvironment in a rat model of chronic vein thrombosis [28].

When DVT occurs, EPCs could home and migrate to the sites of neovascularization, increase new blood vessel formation in the injured site [29], and secrete a variety of vasoactive and angiogenic factors to improve angiogenesis thrombus resolution [25, 30]. In addition, EPCs have the potential to protect differentiated endothelial cells from apoptosis and to preserve their angiogenic capacity under conditions of oxidative stress [31]. The proposed mechanisms of EPCs in thrombus resolution are as follows. EPCs can home and integrate into the site of the injured vessels and thrombi and participate in orchestrating the angiogenesis process. EPCs residing in the thrombi can release a source of paracrine pro-angiogenic factors to induce vascularization. Moreover, EPCs produce proteinases to break down thrombi and secrete other factors to interact with platelets to prevent the apoptosis of mature endothelial cells and the formation of new thrombosis [32].

MiRNAs have been shown to play key roles in vascular development, homeostasis, function, disease and regeneration [33]. In particular, miRNAs have impact on angiogenesis and modulate the behavior of EPCs [15, 34, 35]. Therefore, we proposed that miRNA modified EPCs could improve the efficacy of EPCs in the thrombus resolution and recanalization. However, the potential of EPCs-mediated cancer initiation should be paid attention [36].

MiR-483-3p is located within the insulin growth factor (IGF2) locus at chromosome 11p15.5 and implicated in various human cancers including colon, breast, liver cancers and squamous cell carcinomas [37, 38]. IGF2 is involved in angiogenesis through the regulation of the IGF/IGF2R system, and miR-483-3p was an intronic miRNA within IGF2. Until now, the exact role of miR-483-3p in the function of EPCs has not been determined yet. In the present study, miR-483-5p emerges as a novel angio-miR that negatively regulates angiogenesis. By in vitro tube formation assay, we demonstrated that miR-483-3p modulated angiogenic potential of EPCs. Moreover, we found that miR-483-3p regulated the migration and apoptosis of EPCs.

To elaborate putative mechanism by which miR-483-3p regulates EPCs function, we employed bioinformatics tool and molecular biology method to identify target gene. Our results suggested that SRF could be the potential target of miR-483-3p. SRF is involved in the regulation of multiple genes implicated in cell growth, proliferation, migration, apoptosis, cytoskeletal organization, energy metabolism and myogenesis [39, 40]. Moreover, SRF is important for the appropriate expression of structural proteins such as β-actin, VE-cadherin and several integrins in endothelial cells, and changed expression of SRF could lead to defective vascular morphogenesis [41]. In addition, SRF was reported to mediate FGF signaling in tracheal terminal branching and VEGF/FGF signaling in sprouting angiogenesis [42]. Indeed, previous study reported that miR-483-5p directly targeted SRF in human umbilical vein endothelial cells and inhibited angiogenesis in vitro [43]. Therefore, our present study extended previous findings on miR-483-SRF pathway from endothelial cells to EPCs.

There are some limitations in this study. Our results confirmed that SRF was a target of miR-483-3p and the suppression of SRF by miR-483-3p was involved in the effects of miR-483-3p on EPCs function. However, additional target genes are likely to participate in the anti-angiogenic effect of miR-483-3p, which remain to be investigated in the near future. Furthermore, the criteria of EPCs are still controversial. In this study, we used adherent PBMCs with the features of DiI-ac-LDL endocytosis and UEA-I expression binding, and the cells also showed the expression of CD34, CD133 and VEGFR-2. These EPCs have been shown to self-renew and form tube-like structures in vitro, and there might be several cell subpopulations in these EPCs.

## Conclusions

In conclusion, this study shows that miR-483-3p is upregulated in EPCs from DVT patients and it impairs EPCs function via its target SRF. Furthermore, the application of EPCs transfected with miR-483-3p inhibitor into the vein thrombosis rat model improved the thrombus recanalization and resolution. These data suggest that miR-483-3p might be employed as a therapeutic target in the treatment of thrombus in the clinical practice.

## Additional file

10.1186/s12967-016-0775-2 Identification of differentially expressed miRNAs in EPCs from healthy control and patients with DVT by microarray. The heat map diagram showed the results of the two-way hierarchical clustering of miRNAs and samples (Arraystar, human miRNA 18.0 chip). The color scale shown at the top illustrated the relative expression level of a miRNA in the certain slide: red color represented upregulated miRNAs while green color represented downregulated miRNAs.

## Abbreviations

EPCs: endothelial progenitor cells
DVT: deep vein thrombosis
miRNA: microRNA
SRF: serum response factor
3′-UTR: 3′-untranslated region
GFP: green fluorescent protein
IVC: inferior vena cava
DSA: digital subtract angiography
