# Supplementary figures and images for: Upregulation of miR-483-3p contributes to endothelial progenitor cells dysfunction in deep vein thrombosis patients via SRF
Source: J Transl Med. 2016 Jan 22;14:23. doi: 10.1186/s12967-016-0775-2 (PMC4724160; doi:10.1186/s12967-016-0775-2)

**Fig S1.**


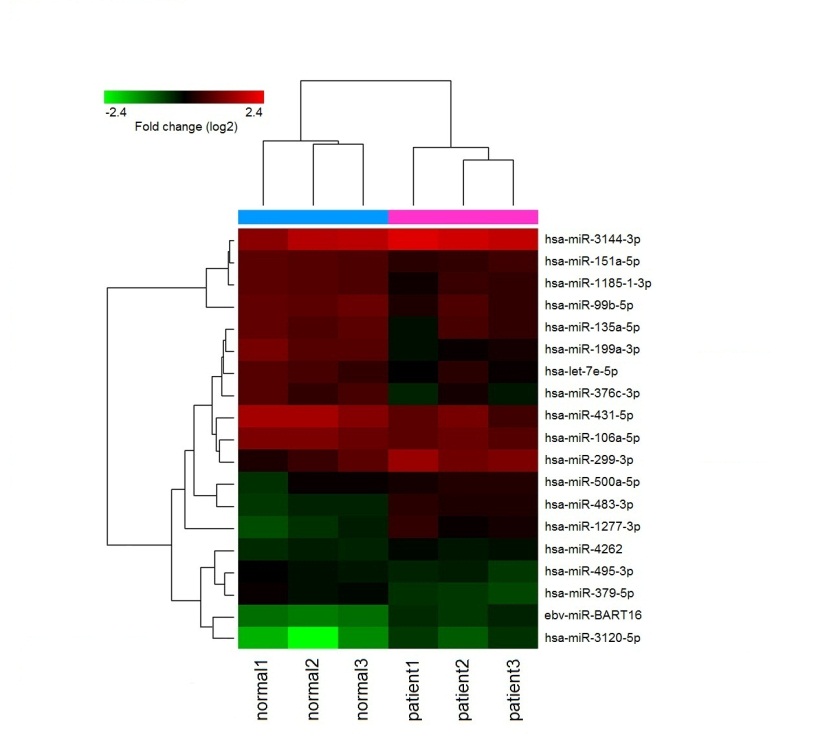

Supplement: Supplementary file 1 — 10.1186/s12967-016-0775-2 Identification of differentially expressed miRNAs in EPCs from healthy control and patients with DVT by microarray. The heat map diagram showed the results of the two-way hierarchical clustering of miRNAs and samples (Arraystar, human miRNA 18.0 chip). The color scale shown at the top illustrated the relative expression level of a miRNA in the certain slide: red color represented upregulated miRNAs while green color represented downregulated miRNAs. [file 12967_2016_775_MOESM1_ESM.doc]
